# Incidence and Risk Factors of Developing Post-operative Delirium Among Elderly Patients in a Tertiary Care Hospital: A Retrospective Chart Review

**DOI:** 10.7759/cureus.65188

**Published:** 2024-07-23

**Authors:** Yasser Almashari, Rand A Alshaya, Radin R Alenazi, Aseel M Alanazi, Reem Alhanan, Futoon A Al-Shammari, Rayan Muawad

**Affiliations:** 1 Medicine, King Saud Bin Abdulaziz University for Health Sciences, Riyadh, SAU; 2 Medicine, Dar Al Uloom University, Riyadh, SAU; 3 Pediatric Anesthesia, King Abdulaziz Medical City Riyadh, Riyadh, SAU

**Keywords:** cross-sectional study, elderly, anesthesia, risk factors, delirium, post-operative delirium

## Abstract

Introduction

Delirium is an acute and fluctuating decline in attention and cognition caused by reversible neural disruption. Post-operative delirium (POD) may happen 10 minutes after anesthesia administration up to discharge. POD has been associated with increased days of mechanical ventilation, increased patients’ functional decline, prolonged intensive care unit (ICU) stay, and prolonged hospital length of stay, which can lead to nosocomial complications, further investigations, and increased treatment costs. In this study, we aim to determine the prevalence of POD and identify demographic or surgical variables associated with POD.

Materials and methods

This retrospective cross-sectional study was conducted at the National Guard Health Affairs Hospital (NGHA), a teaching tertiary care center in Riyadh, Kingdom of Saudi Arabia. The study included all patients older than 65 who developed POD from January 2017 to January 2023 and a control group of the same time window. The data were analyzed using custom Python code.

Results

The study included 108 patients, 72 of whom were male patients. General anesthesia was most used compared to other anesthesia techniques (79.630%). Patients with hypertension (HTN) and diabetes mellitus (DM) each account for 75 cases. Elective surgeries account for 86.111% of cases. Our analysis showed a significant association between POD and advanced age, male gender, DM, HTN, congestive heart failure (CHF), and chronic kidney disease (CKD).

Conclusion

With our study, we hope to aid the process of better understanding POD to help healthcare providers identify high-risk patients, implement preventative measures, and enhance patient safety and satisfaction.

## Introduction

Delirium is an acute and fluctuating decline in attention and cognition caused by reversible neural disruption [1,2]. According to the Diagnostic and Statistical Manual of Mental Disorders, Fifth Edition (DSM-5), the criteria for diagnosing delirium involve a cognitive disturbance that emerges over a brief period and has a fluctuating nature [2]. POD rates vary significantly, ranging from 9% to 87% depending on the age and surgery type [1]. The underlying mechanism of POD is multifactorial since no single factor is responsible for this phenomenon. POD is different from emergence delirium, which occurs after awakening from anesthesia in 8-20% of patients, especially in young patients [3,4].

POD has been associated with increased days of mechanical ventilation, increased patients’ functional decline, prolonged intensive care unit (ICU) stay, and prolonged hospital length of stay, which can lead to nosocomial complications, further investigations, and increased treatment costs [5-8]. A prospective observational study that included 588 patients aged 70 years or older showed that POD is an independent predictor for prolonged hospital stay in the ICU and the hospital [9]. Moreover, POD has been associated with increased morbidity and mortality, as it increases all-cause mortality by at least 10-20% every 48 hours of delirium [10-13]. Furthermore, a systemic review study showed an association between the 10-year probability of death and delirium after cardiac surgeries, and it showed that patients with a history of delirium had an almost twofold increased risk of hospital readmission [14]. Therefore, screening for POD should be performed, especially among vulnerable groups, to minimize its impact.

Predisposing risk factors for developing POD include advanced age, reduced cognitive reserve, atherosclerotic disease, pulmonary disease, renal impairment, and sensory impairment [15]. Precipitating risk factors include using residual neuromuscular blockade, opioid analgesics, anticholinergics, antihistamines, and benzodiazepines [15,16]. 

In this study, we aim to determine the prevalence of POD and identify demographic or surgical variables associated with POD to help healthcare providers identify high-risk patients, implement preventative measures, and enhance patient safety and satisfaction.

## Materials and methods

This retrospective cross-sectional study was conducted at the National Guard Health Affairs Hospital (NGHA), a teaching tertiary care center in Riyadh, Kingdom of Saudi Arabia. The study included all patients older than 65 who developed POD from January 2017 to January 2023. The diagnosis was established by the primary surgical team. Patients who did not develop POD or patients who developed POD but were younger than 65 were excluded from the sample. Furthermore, the study included a control group of all patients older than 65 who did not develop POD within the same timeframe. The hospital database was utilized to collect the sample and control group variables. The collected data were applied in a Microsoft Excel sheet (Microsoft® Corp., Redmond, WA).

A comprehensive statistical analysis was conducted on the dataset using custom Python code, encompassing descriptive and inferential methodologies. Firstly, a descriptive analysis summarized the participants' demographic, medical, and surgical variables, including age, gender, and other features. Statistical testing was conducted to investigate the correlation between the incidence of POD and demographic, medical, and surgical variables. For categorical variables such as gender and anesthesia type, chi-square tests were applied to analyze differences in distribution between groups. Meanwhile, t-tests were utilized to evaluate differences in continuous variables, such as age, thereby highlighting its influence on the risk of developing postoperative delirium. A significance threshold (α) of 0.05 was adopted for all tests, carefully balancing the risk of type I and II errors.

Given the potential for type I error inflation due to multiple testing, we adopted the Benjamini-Hochberg False Discovery Rate (FDR) control to adjust our findings, thus ensuring reliability amidst the exploration of multiple risk factors. This adjustment method was instrumental in acknowledging the interconnectedness of the variables under study and their collective impact on the outcome of interest.

## Results

Table 1 demonstrates all the descriptive data of our sample. The study included 108 patients, 72 of whom were male patients. Furthermore, the age range was from 65 to 101. The mean age is approximately 76.87 years, with a standard deviation of 7.166 years. For anesthesia types used in surgeries, general anesthesia is the most common (79.630%), followed by topical, nerve block, monitored anesthesia care, and epidural in descending order of frequency.

**Table 1:** Demographics and clinical variables of the sample

Variable		Count	Frequency (%)
Gender	Male	72	66.667
	Female	36	33.333
Age (years)	65-70	23	21.296
	71-80	63	58.333
	81-90	18	16.667
	91-100	3	2.778
	101,110	1	0.926
Type of post-operative delirium	Unspecified delirium	79	73.1
	Delirium of mixed origin	29	26.9
Anesthesia type for surgery	General	86	79.6
	Topical	12	11.1
	Nerve block	5	4.6
	Monitored anesthesia care	3	2.7
	Epidural	2	1.9

Table 2 shows the common comorbidities among delirium patients, with hypertension (HTN) and diabetes mellitus (DM) each accounting for 75 cases. Congestive heart failure (CHF) is next, with 45 cases. Chronic kidney disease (CKD) has 26 cases.

**Table 2:** Comorbidity in patients who experienced post-operative delirium (n=108)

Co-morbidity	Count	Frequency (%)
Diabetes mellitus	75	81
Hypertension	75	81
Congestive heart failure	45	48.6
Chronic kidney disease	26	28.08

Table 3 shows delirium incidence across surgical variables. Elective surgeries account for 86.111% of cases, while emergent cases represent 13.889%. The most common surgery type in the sample is orthopedic surgery, followed by vascular surgery.

**Table 3:** Delirium incidents across various surgical contexts

Variable		Count	Frequency (%)
Case type	Elective	93	86.111
	Emergency	15	13.889
Surgery specialty	Abdominal surgery	11	10.185
	Orthopedic surgery	23	21.296
	Cardiac surgery	12	11.111
	Urology surgery	7	6.481
	Neurosurgery	15	13.888
	Ophthalmologic surgery	2	1.852
	Anorectal surgery	3	2.777
	Urology diagnostic procedure	12	11.111
	Vascular surgery	17	15.741
	Head and neck	2	1.852
	Plastic surgery	4	3.704

For the data analysis, we integrated data from two distinct groups: all patients who are 65 years or older who developed POD within the specified time frame of the study and patients who are 65 years or older who did not develop POD. This integration involved aligning demographic and clinical variables, including gender, age, and medical history elements such as hypertension and diabetes. Employing descriptive statistics, we gained insights into the demographic distribution and prevalence of conditions within our dataset, setting the stage for deeper analysis.

Our analysis (Table 4) revealed a statistically significant association between gender and the occurrence of postoperative delirium, with a higher proportion of males in the sample group. This was substantiated by a chi-square statistic of 7.003 and a p-value of 0.0081, indicating a noteworthy gender-based difference in the incidence of delirium. Moreover, our analysis revealed a significant difference in the mean age between the sample and control groups, highlighted by a T-statistic of 13.365 and a p-value of 1.15e-40, underscoring age as a critical factor in delirium risk. In contrast, our investigation into the influence of general anesthesia did not demonstrate a statistically significant association with postoperative delirium, as indicated by a chi-square statistic of 0.0095 and a p-value of 0.922, suggesting that anesthesia type might not significantly impact delirium risk within our study's scope.

**Table 4:** Inferential statistics of patients’ variables and POD POD: post-operative delirium. DM: diabetes mellitus. HTN: hypertension. CHF: congestive heart failure. CKD: chronic kidney disease

Variable	P-value
Gender association with POD	0.0081
Age difference between groups	1.15e-40
General anesthesia association with POD	0.922
DM association with POD	6.38e-10
HTN association with POD	3.81e-12
CHF association with POD	2.12e-112
CKD association with POD	1.16e-25

Furthermore, the association between DM and postoperative delirium was statistically significant, with a chi-square test yielding a p-value of 6.38e-10, suggesting a higher prevalence of DM in the sample group. A significant link between HTN and postoperative delirium was established, with a p-value of 3.81e-12, indicating a greater prevalence of HTN among those in the sample group. CHF was profoundly associated with postoperative delirium, as evidenced by a chi-square test result with a p-value of 2.12e-112, underscoring a substantial correlation. The relationship between CKD and postoperative delirium was also significant, with a chi-square test producing a p-value of 1.16e-25, pointing to a higher occurrence of CKD in the sample group.

All in all, our analysis showed a significant association between POD and advanced age, male gender, DM, HTN, CHF, and CKD. 

## Discussion

Our study cohort revealed a significant association between male gender and POD, which aligns with the existing literature [17-19]. Edelstein et al. showed that male patients had double the incidence of POD than female patients [20]. This may be because the male gender is considered a risk factor in neuropsychiatric problems, while estrogen is a protective factor [17. Nevertheless, the exact mechanism is unclear, and further investigations are needed.

Moreover, our analysis demonstrated that age was significantly associated with POD. Age is a known risk factor for POD. A previous study showed that the occurrence of POD in patients aged 50-59 was 22%, and in patients aged 80-89, it was up to 92% [21]. This might be because of the association between advanced age and decreased overall cognitive abilities.

It is generally believed that GA exposure is associated with an increased risk of POD, but the literature is still conflicted in this regard [22-25]. A meta-analysis by Ko et al. showed that general anesthesia exposure was associated with an elevated risk of POD with a P-value of 0.0003 [26]. However, a large-scale randomized controlled trial did not find any difference in the incidence of POD between GA and spinal anesthesia in two randomly assigned groups [27]. Our study showed general anesthesia was not associated with an increased incidence of POD.

Regarding comorbidities, DM, HTN, CHF, and CKD were significantly associated with POD in our cohort. DM has been consistently identified as a risk factor for delirium due to its association with cognitive dysfunction. Ganai et al. showed that hyperglycemia is a risk factor for developing in-hospital delirium in geriatric patients undergoing abdominal surgery [28]. Kotfis et al. showed that patients with delirium were more likely to be diabetic and on oral diabetes drugs, and there was no difference in patients on insulin treatment [29]. Hyperglycemia induces inflammation on a cellular level, and endogenous or exogenous insulin normalizes glycemia and is also anti-inflammatory [30,31]. Therefore, tight control with insulin treatment would reduce the risk of POD.

Hypertension showed a high correlation with POD, with 81% of the population being diagnosed with HTN; this finding also correlates with a study conducted by Weinstein et al., which concluded that 61.4% out of 922 patients who were diagnosed with POD of patients who did total knee and arthroplasty had HTN [24]. CHD was also identified in 48.6% of our patients, which also correlates with Parente et al.'s study that documented that 50% of the diagnosed cases had CHF [32] but contraindicates Weinstein et al. findings [24]. Chronic kidney disease was the least comorbidity among our population, with a percentage of 28.08%. Kotfis et al. and Parente et al.'s studies also showed similar results of CKD being the least common condition among patients diagnosed with delirium, with rates of 12.55% and 17%, respectively. This may be because CKD, compared to the other listed comorbidities in our study, is less seen when we compare them to the populations with DM and HTN [24,32].

There are several limitations to our study. Firstly, the sample population was taken from only one region (Riyadh, Saudi Arabia), limiting our results' representativeness. Secondly, we could not add any management to the study due to the lack of information that could have been useful to the readers or any new studies in the future to compare the management between our study and theirs. Thirdly, unfortunately, any medication given perioperatively could not be obtained from the software, which could have helped give us more information on what medications are associated with developing POD.

## Conclusions

With our study, we hope to aid the process of better understanding POD and provide a resource for future studies to understand POD further. Understanding POD is crucial for screening and prevention, especially considering this condition's health, emotional, and financial burden to the patients.
